# Supplementary material for: Carnitine insufficiency is associated with fatigue during lenvatinib treatment in patients with hepatocellular carcinoma
Source: PLoS One. 2020 Mar 3;15(3):e0229772. doi: 10.1371/journal.pone.0229772 (PMC7053710; doi:10.1371/journal.pone.0229772)
Supplement: Attachment — Submitted filename: Response to Reviewers OKUBO.rtf [file pone.0229772.s002.rtf]

Response to the Editor

11 January 2020
Dr. Tatsuo Kanda
Academic Editor
PLOS ONE

Please find attached a copy of our revised manuscript entitled “Carnitine insufficiency is associated with fatigue during lenvatinib treatment in patients with hepatocellular carcinoma”, which we would like to resubmit for publication in PLOS ONE. 

We would like to thank the academic editor and the reviewers for their helpful comments. We have implemented the suggested changes and trust that our manuscript is now suitable for publication in your journal. Please find below a detailed description of the revisions made to the manuscript in our point-by-point responses to the comments from the reviewers.

Yours sincerely,

Hironao Okubo, MD, PhD 
Department of Gastroenterology 
Juntendo University Nerima Hospital   


Response to academic editor

1)	Authors should describe the status of HBsAg and anti-HCV in table 1, and discuss their influence.

Response: Thank you for your valuable comments. In accordance with the comment, we have added information about the etiology, which includes the presence or absence of HBs-Ag/HCV-Ab, in Table 1 and the Discussion section (lines 256–258 as follows:
There was a strong correlation between BFI and AC/FC ratio at day 28 existed regardless of the presence or absence of viral hepatitis, whether it was a viral infection or other causes (r = 0.840 and r = 0.773, respectively).

2)	Major problem is that the number of patients is too small, and authors should increase the number of patients to at least 20.

Response: In accordance with the advice, we added the two most recent cases, and the data from 20 patients were reanalyzed. The revised results further support our conclusion.

#Response to reviewer 1
1)	In Figure 4, the authors showed the correlation between BFI and AC/FC ratio in Day 14, and Day 28. Please add the figure of the correlation between BFI and AC/FC ratio at baseline. If correlation was not showed at baseline, please discuss the cause of this phenomenon.

Response: In accordance with the comment, we added the data for the correlation between BFI and the AC/FC ratio at baseline to Figure 4 of the revised manuscript. We found that there was a nearly significant positive correlation between the two variables (Fig 4A; r = 0.410, p = 0.073). This description was added to the revised manuscript (lines 200-202).

2)	Please insert reference to discussion section, line 256.

Response: In accordance with the reviewer's advice, we have added the following reference to the Discussion (line 281 in the revised manuscript): Takahashi A, Moriguchi M, Seko Y, Ishikawa H, Yo T, Kimura H, et al. Impact of relative dose intensity of early-phase lenvatinib treatment on therapeutic response in hepatocellular carcinoma. Anticancer Res. 2019;39:5149-56. 

#Response to reviewer 2 
1.	As the authors mentioned in the limitations, the study cohort is too small. The reviewer would suggest that the authors evaluate ATP or energy charge level and/or OCTN functions of the patients. Detailed analysis using more parameters or translational experiments might add strength in the study regardless of the cohort size.

Response: Thank you for the insightful and constructive comment. We agree with the comment, but evaluation of ATP/energy charge level or OCTN functions is extremely difficult in patients with cancer in clinical practice. Therefore, in accordance�@with the academic editor's advice, we added the additional two cases and re-analyzed the data for all 20 patients. We believe that our present clinical data are valuable and worth publishing in PLOS ONE.


2.	The authors should provide information about treatment response and its relationship with the carnitine levels.

Response: In accordance with the comment, we have conducted an additional analysis about the relationship between the treatment response and carnitine levels. 

The treatment responses were added to Table 1, and we discussed the relationship in the revised manuscript (lines 305–310) as follows: In addition, it is important to consider the type of patients who are recommended to receive carnitine supplementation. Because the baseline plasma AC/FC ratio in non-responders (SD and PD) tended to be higher compared with that of responders (CR and PR) (p = 0.057), it might be better to consider levocarnitine supplementation in patients with a high AC/FC ratio to retain the relative lenvatinib dose intensity. 


3.	The comparison of AC/FC levels between sorafenib and lenvatinib would be of interest. 

Response: We thank the Reviewer for this insightful comment. In accordance with this comment, we added the presumptive difference in the inhibitory effect on OCTN2 between lenvatinib and sorafenib, which would affect the AC/FC level, into the Discussion section of the revised manuscript (lines 275–278) as follows: However, because more fatigue was reported during lenvatinib therapy compared with sorafenib [3], it is possible that lenvatinib's inhibitory effect on OCTN2 is stronger compared with that of sorafenib.

4.	Although the BFI improved after the supplementation, AC/FC did not decrease. The authors conclude the well correlation between AC/FC and BFI during the treatment and they should discuss why this happened (why the AC/FC did not improve after the supplementation).  

Response: We would like to express our strong appreciation to reviewer 2 for this comment. As the reviewer pointed out, the AC/FC ratio did not necessarily improve after the levocarnitine supplementation. We think that the placebo effect, including the recognition of dose reduction, might influence the BFI score especially in some patients with dose reduction. We provided this interpretation on lines 296–298 of the revised manuscript. Moreover, for reader's convenience, we have changed Figure 5, which separately shows patients with and without dose reduction. 


#Response to Journal Requirement
1.	We have checked that our manuscript meets STROBE statement.

2.	a) As pointed out, the notation has been changed as follows: This study was approved by the Ethics Review Board of Juntendo University Faculty of Medicine (Jundai-Irin No-2016135) and was performed in accordance with the 1964 Declaration of Helsinki and its later amendments.
b) In accordance with the comment, we have inserted the same text to the “Ethics Statement”.

3.	Thank you for pointing some minor occurrences of overlapping text. The notation has been changed as follows. 

Lines 68–69: Patients with cancer occasionally experience fatigue and general malaise before and after treatment, which is described as cancer-related fatigue.
Line 98–99: This study was approved by the Ethics Review Board of Juntendo University Faculty of Medicine
Line 185–189: The box spans data between two quartiles (IQR, interquartile range), and the bold line represents the median. The ends of the whiskers represent the largest and smallest values that are not outliers.
