# Supplementary material for: Carnitine insufficiency is associated with fatigue during lenvatinib treatment in patients with hepatocellular carcinoma
Source: PLoS One. 2020 Mar 3;15(3):e0229772. doi: 10.1371/journal.pone.0229772 (PMC7053710; doi:10.1371/journal.pone.0229772)
Supplement: Attachment — Submitted filename: renamed_31884.rtf [file pone.0229772.s003.rtf]

Response to the Editor

3 February 2020
Dr. Tatsuo Kanda Academic Editor
PLOS ONE

Dear Dr. Kanda,

Please find attached a copy of our revised manuscript titled “Carnitine insufficiency is associated with fatigue during lenvatinib treatment in patients with hepatocellular carcinoma”, which we would like to resubmit for publication in PLOS ONE. 

We would like to thank the reviewer for the helpful comments. We have implemented the suggested change and trust that our manuscript is now suitable for publication in your journal. Please find below a detailed description of the revision made to the manuscript in response to the comment from the reviewer.

Yours sincerely,

Hironao Okubo, MD, PhD 
Department of Gastroenterology 
Juntendo University Nerima Hospital   


#Response to reviewer 2 
1.	In the study, authors measured urine/plasma AC/FC. The reviewer was wondering if they could describe which marker was the most useful in the real-world clinical setting: the reason, speculation, and how often it should be monitored. Please add this information in the discussion section.

Response: Thank you for the insightful comment. In accordance with the comment, we discussed the utility of the plasma AC/FC ratio in the real-world clinical setting in the revised manuscript (lines 311–316) as follows: Throughout the study period, only the plasma AC/FC ratio among all of the markers (plasma FC, plasma AC, urine FC, urine AC levels, and urine AC/FC ratio) was associated with BFI (data not shown). Therefore, only the plasma AC/FC ratio seems to be a useful marker during lenvatinib therapy. As mentioned above, the baseline plasma AC/FC ratio seems to be useful for predicting incomplete treatment with lenvatinib.
