# Supplementary material for: Carnitine insufficiency is associated with fatigue during lenvatinib treatment in patients with hepatocellular carcinoma
Source: PLoS One. 2020 Mar 3;15(3):e0229772. doi: 10.1371/journal.pone.0229772 (PMC7053710; doi:10.1371/journal.pone.0229772)
Supplement: Attachment — Submitted filename: R3 Response to the Editor.rtf [file pone.0229772.s004.rtf]

Response to the Editor

12 February 2020
Dr. Tatsuo Kanda, Academic Editor
PLOS ONE

Dear Dr. Kanda,

Please find attached a copy of our revised manuscript titled “Carnitine insufficiency is associated with fatigue during lenvatinib treatment in patients with hepatocellular carcinoma”, which we would like to resubmit for publication in PLOS ONE. 

We would like to thank for the helpful comment. We have added a supplementary file including the patient characteristics and data, and we trust that our manuscript is now suitable for publication in your journal. 

Yours sincerely,

Hironao Okubo, MD, PhD 
Department of Gastroenterology 
Juntendo University Nerima Hospital   


#Response to the academic editor 
1.	Authors should make a list of each patients' characteristics and data by an excel file and submit the excel file as a supplementary file with manuscript again.

Response: Thank you for the helpful comment. In accordance with the comment, we have added a supplementary file including the patient characteristics and data, and we trust that our manuscript is now suitable for publication in your journal. In addition, we have added the supporting information into the revised manuscript (lines 330–331) as follows: 

Supporting information
S1 Data. Patient characteristics and data.
